# New products

**DOI:** 10.1155/S1463924685000128

**Published:** 1985


Journal of Automatic Chemistry, Volume 7, Number 1 (January–March 1985), pages 50–56

### New products

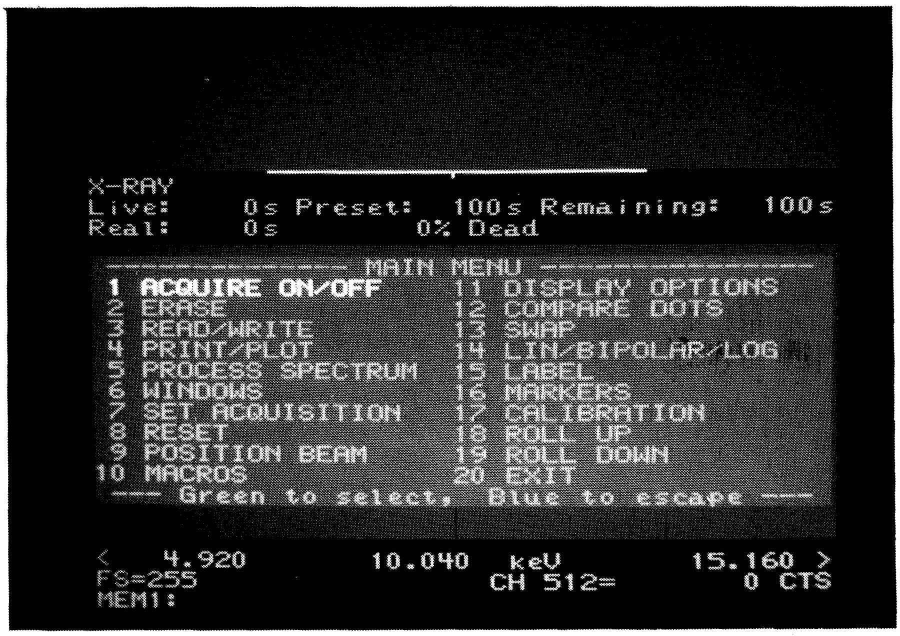

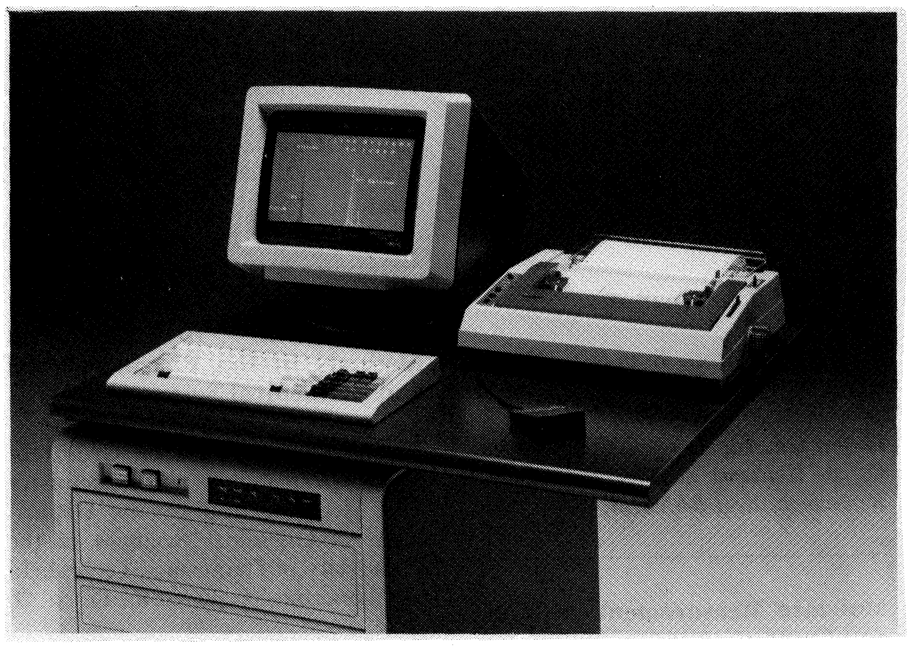

*The AN10000 X-ray microanalysis system—console and activities menu. The manufacturer, Link Systems, has been established as one of the World's leading manufacturers of X-ray microanalysis and X-ray fluorescence equipment for over a decade. Based in High Wycombe, it exports much of the advanced high-technology equipment it manufactures.*

### X-ray microanalysis system

A new computer-based X-ray microanalysis system has been launched by Link Systems. The all-British set-up is claimed to be the most technologically advanced of its type currently available world-wide. It's called AN10000 and is capable of measuring, storing, analysing, displaying and manipulating X-ray and electron signals produced by scanning and transmission electron microscopes. The technique is applicable in such fields as chemical analysis, semiconductor research and quality-control, metallurgy and forensic science.

Features include a microcomputer, designed by the manufacturer, which controls data-handling operations. The machine can outperform most of the micros currently on the market. The basic 256K central processor operates within a purpose-designed disk-operating system. The AN10000 is the first such system to incorporate Winchester disks as a standard feature: each disk, supported by a back-up floppy disk, provides up to 32 times the on-line data-storage capacity of floppy-disk based systems.

An attraction is a high-resolution colour monitor, used in conjunction with a keyboard and 'mouse'. Interaction between the user and system is through a series of displayed screen menus, and selection from these is made either through the keyboard or the mouse. By simply moving the mouse around on a flat surface, images or cursor may be moved to enhance, clarify and speed up selections.

Hard copy may be printed-out with a built-in matrix printer/plotter. The system offers 'screen copy' facilities, and data from quantitative analyses can be produced immediately in a quality suitable for reproduction and inclusion in reports.

Link Systems believe that the most important feature of the AN10000 is its operating software. The company has, over the past decade, developed many software packages for specific analysis tasks and the AN10000 will accept them all. A new package is 'SCREED': for word-processing.

Software is user interactive and may be modified to each user's requirements using Link's FORTRAN package.

A modular design allows the keyboard and display to be positioned remotely from the main console and configured to the user's needs.

A full range of associated high performance Si(Li) detectors are available. The new Series E detectors, coupled with a time-variant filter pulse processor line, provide excellent spectral stability.

*More information from Link Systems Ltd, Halifax Road, High Wycombe, Buckinghamshire HP12 3SE, UK. Tel.: 0494 442255.*

**Circle No. 1 on Reader Enquiry Card**

### Applications for LDC/Milton Roy detector

The recently launched e.c. Monitor amperometric detector now has applications data available in the form of seven 'Technical notes'. One general note looks at 'Candidate compounds for determination by liquid chromatography and amperometric detection'; the others are specific in the determination of benzidine, urinary catecholamines, chlorinated phenols and ascorbic acid in food products, pharmaceuticals and human urine. Additional notes are planned.

*Copies from Laboratory Data Control (UK) Ltd, Milton Roy House, High Street, Stone, Staffordshire ST15 8AR, UK. Tel: 0785 813542.*

**Circle No. 2 on Reader Enquiry Card**

### Plasma spectrometer

The ICP/6500 is Perkin-Elmer's latest sequential, inductively coupled plasma emission spectrometer. The system is capable of analysing trace elements routinely at a speed of over 20 elements/min and of determining samples at any wavelength without prior wavelength calibration. Analyses are performed at the optimum analytical wavelength for each element or sample matrix, providing unsurpassed analytical flexibility.

The ICP/6500 incorporates all the features of its predecessor, the ICP/6000, and includes new software and a new plasma torch and power supply.

The high-performance optical system uses a dual-grating monochromator covering the wavelength range 170–900 nm. A blazed holographic grating is used in the ultraviolet region of the spectrum and each grating is used only in the first order. These factors ensure efficient light handling and low stray light. In addition, the optical system can be purged with an inert gas, so analyses can be performed routinely below 190 nm without purchasing additional components.

The new plasma torch assembly and the compact RF power-supply of the ICP/6500 were designed for stable and reliable operation. The torch is demountable to reduce maintenance costs, and the complete sample-introduction system is resistant to attack from corrosive sample solutions: strong acids can be aspirated and high concentrations of dissolved solids are also handled easily.

The ICP/6500 is controlled through a Perkin-Elmer 7300 Professional Computer (a 16-bit microcomputer with a 15 megabyte Winchester hard disk and unlimited data archiving on floppy disk). High-resolution colour graphics allow spectra to be compared and evaluated easily, since standards, blanks and samples are displayed in their own designated colours. Analytical results are stored as they are generated on the hard disk and reformatted later to produce customized reports on a multicolour graphics printer.

Bezel-mounted software programmed keys simplify parameter entry for method development. For quantitative multi-element analysis, methods can be built containing up to 108 wavelengths, or the Qmode permits semi-quantitative analysis of a sample by using stored standardization information. The system contains on-line listing of over 50 000 analytical wavelengths for rapid identification of unknown emission peaks and wavelength referencing. Diagnostic and instructional routines are also included.

*For further information contact Perkin-Elmer Ltd, Post Office Lane, Beaconsfield, Buckinghamshire HP9 1QA, UK. Tel.: 04946 6161.*

**Circle No. 3 on Reader Enquiry Card**

### Oil 'fingerprinting'

A Beckman SpectraSpan plasma emission spectrometer is now in use by hydrocarbon analysts T. J. Gunner & Sons Ltd of Burgess Hill, Sussex, UK. It performs a variety of tasks in the analysis of oil and the detection of minute quantities of elements.

T. J. Gunner & Sons are also marine surveyors and are called upon when there is an oil leak at sea to analyse samples of the spill with a view to identifying its source by means of various techniques, including elemental analysis – a 'fingerprinting' technique using the SpectraSpan.

Analysis of all kinds of oil is carried out using the SpectraSpan in Gunner's laboratories – sea pollution with oil is just one aspect of its work. A contract has been obtained, for example, for the analysis of aircraft oils and there is frequently a need to test oil additives to see whether they have been broken down due to age or to detect contaminants in oil samples. The use of the SpectraSpan with its high accuracy and repeatability is important in these techniques, as well as for the detection of lead in gasoline which enables the laboratory to report the percentage of contamination in diesel fuel.

*Details about SpectraSpan from Beckman-RIIC Ltd, Progress Road, Sands Industrial Estate, High Wycombe, Buckinghamshire, UK. Tel.: 0494 41181.*

**Circle No. 4 on Reader Enquiry Card**

### Laboratory automation in High Wycombe

Following the recent formation of Laboratory Automation Operations by Beckman in the USA, the company has now established a European Centre for Laboratory

Automation with headquarters in the UK at High Wycombe.

John Boother, a chemist, has been appointed Manager of the new Centre. He previously worked for 12 years with Hewlett-Packard and, more recently, was UK Sales Manager of the Analytical Division of Kontron.

The product range consists of specialized, fully supported computer programs which run on hardware from several manufacturers. At present, Beckman offers programs for the collection and manipulation of data from toxicology experiments (TOXSYS), the search of organic synthesis data-bases to assist synthetics chemists (SYNLIB), the collection and storage of data from analytical instruments either on personal computer or microcomputer, and full laboratory data management (CALS).

Also based at High Wycombe are two Technical Support Specialists responsible for providing software support to customers by means of demonstrations, installation, training and telephone advice.

*More information from Beckman (above).*

**Circle No. 5 on Reader Enquiry Card**

### Christian Rovsing take-over

The Industrial Division of Christian Rovsing was acquired in Autumn 1984 by Novo Industri and Superfos for Dkr. 10·7 M. The Division is to be an independent company towards which Novo and Superfos will each contribute a half of the Dkr. 6 M capitalization.

The Rovsing Industrial Division develops and markets Procos Process Control Systems, customized hardware and software packages for monitoring and control of food processing and chemical and pharmaceutical process units. Both Novo and Superfos currently use the Procos system and have entered into the joint acquisition of Rovsing's Industrial Division partly to ensure continued support of their internal systems.

Superfos is one of the largest industrial groups in Denmark. It includes over 50 companies and more than 125 plants and factories. Superfos produces fertilizers, grain and feedstuffs, chemicals, road construction and insulating materials. The Group employs about 5000 people; annual turn-over is around Dkr. 9000 M.

Novo Industri is the world's largest producer of industrial enzymes and the second largest manufacturer of insulins. Headquartered in Bagsvaerd, Denmark, Novo markets its products in 120 countries and maintains research and production facilities in Denmark, Switzerland, France, South Africa, Japan, Canada and the United States.

*More information from Novo Industri A/S, Novo Allé, 2880 Bagsvaerd, Denmark.*

**Circle No. 6 on Reader Enquiry Card**

### Ingold in the UK

The United Kingdom distributor of Ingold products, from September 1984, is Life Science Laboratories Ltd. Ingold pH, $dO_2$ and $CO_2$ electrodes are particularly specified for fermentation processes. The range has been popular since 1947, when Dr W. Ingold invented the combined pH electrode; applications include high pressures, steam sterilization and flow-through with gel-filled, ion-selective and micro-electrodes. Life Science Laboratories have pointed out that all Ingold products sold through them are the genuine Swiss product and should not be confused with either the previously available items manufactured here under licence or with electrodes being offered to fit Ingold probes by other suppliers.

*Life Science Laboratories are in Sarum Road, Luton LU3 2RA and technical support is available on 0582 597676.*

**Circle No. 7 on Reader Enquiry Card**

### PrepSep

A new disposable extraction column which speeds up sample clean-up for HPLC, GC, TLC and UV analyses. Developed by Fisher, the device is designed to replace a variety of traditional preparation techniques, including liquid-liquid extraction. PrepSep isolates and concentrates samples.

Its *conical* design lets the user nest the column inside a centrifuge tube (then control the rate and amount of sample flow-through simply by adjusting time and rpm of centrifugation). Or a vacuum device or hand-held syringe can be used to pull the sample through.

PrepSep is all-polypropylene, with a 10 ml 'reservoir' at the top for ease of use. The *packing*, 300 mg of $C_{18}$ or silica, with high-activity surface area, is sandwiched between two polyethylene frits of 20 μm pore. The packing itself has a pore size of 40 μm.

In addition to its $C_{18}$ and silica models, Fisher plans to announce columns with $C_1$, $C_8$, CN (cyano) and amino packings.

*Further information from Fisher Scientific, 711 Forbes Avenue, Pittsburgh, Pennsylvania 15219, USA. Tel.: 412 562 8468.*

**Circle No. 8 on Reader Enquiry Card**

### Thin film deposition

A new deposition source enables co-deposition and conformal coatings to be achieved from the same source. Up to three of the low-pressure high-rate magnetrons developed by Ion Tech Ltd are combined in this one source to enable binary and ternary alloys or compounds to be deposited.

Replacing one of the magnetrons by Ion Tech's versatile B100WF 'atom beam' gun allows substrate surfaces to be pre-cleaned without charge – induced damage; deposition can then proceed with the substrate irradiated by the neutral atom beam. Energy imparted to the surface in this way removes foreign atoms, produces higher packing densities and redistributes material onto the side walls of any etched features.

The emission of each magnetron can be varied independently of the others, allowing control over alloy composition and enabling concentration gradients to be achieved. D.C.

or R.F. power supplies give total flexibility in target materials.

Oxides, nitrides etc. can be sputtered from an R.F.-powered magnetron and stoichiometry correcting gas supplied via the 'fast atom' gun – which operates on the most reactive gases – this technique offers significant advantages in the precise control of dopant gases.

*For further details contact Mr E. G. Mihill, Ion Tech Ltd, 2 Park Street, Teddington, Middlesex TW11 0LT, UK. Tel.: 01 977 8275.*

**Circle No. 9 on Reader Enquiry Card**

### High-speed motion analysis

The NAC Model HSV-200 high-speed video recording system (I.I.M.C. Ltd) and the Hadland Mekel 300 high-speed camera system are available on hire in the UK.

The HSV-200 records at 200 pictures/s, with exposure times as short as 1/50 000th of a second, using the specifically designed synchronized strobe lights supplied. A 500 ms rotary shutter is built into the colour video camera, enabling use in ambient light conditions. Up to 1 h continuous recording with auto repeat mode operation is available using standard VHS tape in either colour or black-and-white. The 14 in high precision colour monitor gives immediate stable replay in normal, slow motion, still, step and reverse modes selected either from the VTR front panel or the remote control unit. Individual frames are time-referenced, enabling calculation of velocity and acceleration of moving parts. In addition, two audio tracks are included for cue signals and live commentaries, either during or after video recording.

The Mekel 300 uses polavision phototape film-cassettes and the operating speed is digitally selectable from 4 to 300 frames/s. It features 'through the lens' light metering with red, green and yellow LED indicator lights. High power, robust, air-cooled tungsten halogen lamps with free-standing mounts are provided. Automatic film processing and replay is achieved in a back projecting Polaroid polavision analyser, results being fully available 90 s after insertion of the exposed cassette. Replay speed is selectable from a remote hand-held control giving two, four, six and nine frames/s, also single frame forward and fast reverse. Films are available in black-and-white or colour and the sealed cassette format eliminates problems of film threading and chemical handling.

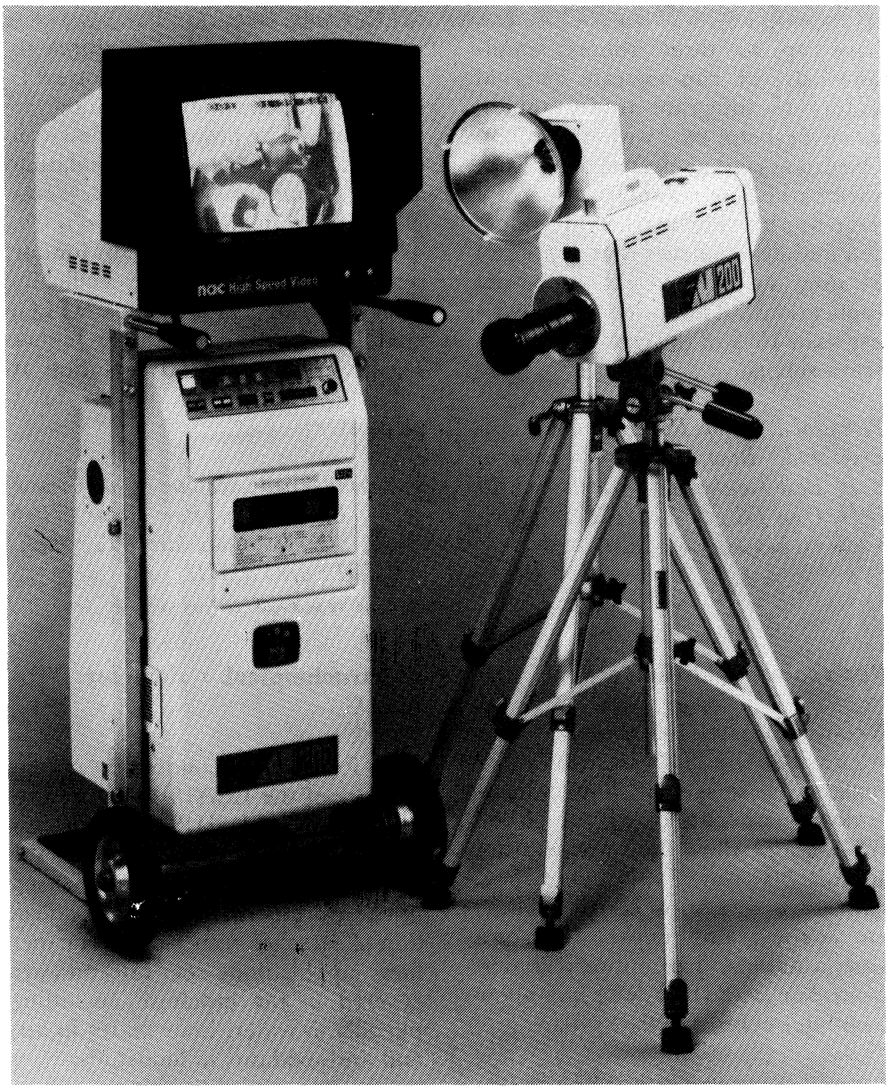

*The HSV-200 high-speed video system available on hire in the UK from Auriema – an operator is provided for daily or weekly rentals. The other high-speed motion analysis machine that the company are hiring out is the Mekel 300, which can be taken with or without an operator.*

Both of these systems have wide applications in industry for fault-finding, machine development and upgrading of high-speed equipment used in, for example, packing, wrapping and capping machinery, food industry, paper and printing industry, engineering and manufacture.

*Details of the hire service from Auriema Ltd, 442 Bath Road, Slough, Berkshire SL1 6BB, UK. Tel.: 06286 4353.*

**Circle No. 10 on Reader Enquiry Card**

### Japanese-type robot

A British company, Reekie Research, has developed a Scara-type robot: this is the dominant form of robot used in Japan and now accounts for the largest single category in the world robot population. Aimed at teaching students the principles of Japanese industrial robots the unit is called *Cepek*, after Karel Cepek who first popularized the word 'robot'. *Cepek* has five degrees of freedom, can lift 4 lb and operates from any microcomputer. The electronics can drive up to three robots simultaneously, or can operate external motors to integrate rotary tables, conveyor belts etc. with the robot. The drive is either electric or electric/pneumatic. The manufacturer sees a large world-wide market in education and also believes that *Cepek* can be used in light-weight industrial applications. The price is £2995 – less than 10% of the cost of previous Scara robots.

*Technical data from Reekie Research Company Ltd, Beaufort Works, Beaufort Road, East Twickenham, Middlesex TW1 2PH, UK. Tel.: 01 892 2877.*

**Circle No. 11 on Reader Enquiry Card**

### Recirculating chiller

A continuous, reliable cooling system is provided by FTS Systems's RC coolers. These systems have a heat-removal capacity of up to 60 000 BTU/h and allow fluid temperature control between −40 °C to +75 °C with an accuracy of ±0·5 °C. These are coupled with one of the company's pumps. The optional digital controller and IEEE/488 bus allow computer control of the system.

*For more information request FTS Systems Inc.'s Bulletin RC: Route 209, P.O. Box 158, Stone Ridge, New York, USA. Tel.: 914 687 7664.*

**Circle No. 12 on Reader Enquiry Card**

### Flow-cell options

A range of flowcells for the uvMonitor series of fixed wavelength detectors and the spectroMonitor variable wavelength detectors is now available from LDC/Milton Roy. Flowcells are available in conventional (analytical) HPLC, preparative, semi-preparative, and microbore LC configurations. The flow-cell accessory kits are designed to be easily interchanged with existing cells. Two types of analytical flow-cells are available, i.e. a 1000 psi flow-cell and the maxN series high pressure/high efficiency flow-cell (pressure rated to 1000 psi). The semi-preparative flow-cell applications are usually related to the isolation of purified components from complex mixtures in intermediate concentrations or samples that require high resolution for separation. The preparative flow-cell option is effectively used for isolating large amounts of purified components with high solvent flow rates. The microCell (spectroMonitor series detectors only) is specifically designed for microbore HPLC, enabling the user to achieve higher sensitivity and greater resolution at very low flow rates (down to 1 µl/min).

*Full details from Laboratory Data Control (UK) Ltd, Milton Roy House, High Street, Stone, Staffordshire ST15 8AR, UK. Tel.: 0785 813542.*

**Circle No. 13 on Reader Enquiry Card**

### Electrochemical detector for HPLC

EDT Research of London, the manufacturer of the LCA15 Electrochemical detector for HPLC, have just released a new brochure on the instrument. It is already widely used in clinical laboratories for the detection of catecholamines. Some further new applications of interest are covered in the brochure, including phenols in water, isocyanates in air, and anti-oxidants in foodstuffs.

*Copies from EDT Research at 14 Trading Estate Road, London NW10 7LU.*

**Circle No. 14 on Reader Enquiry Card**

### Protein evaluation by HPLC

Characterization of proteins and their hydrolysates in terms of molecular weight distribution is one of the most important jobs in Croda Colloids's analytical laboratory.

Gel filtration and gel electrophoresis are techniques already used by the laboratory. The recent introduction of Beckman HPLC systems has provided a way of evaluating protein samples much more rapidly and economically than was previously possible: typically up to six runs per day instead of the previous two or three each week. It is intended that the Beckman HPLC equipment – consisting of a simple isocratic system with a 160 UV detector – will be used additionally in the laboratory for specific amino-acid determinations, as well as a general analytical system.

Croda Colloids Ltd is a major manufacturer of gelatin for the food, pharmaceutical and photographic industries and also produces a range of speciality protein extracts, hydrolysates and chemically-modified derivatives for the cosmetic industry. The Analytical Laboratory on the Ditton site at Widnes provides both a quality-control function and an analytical support service for R&D.

*More information from Beckman Ltd, Progress Road, Sands Industrial Estate, High Wycombe, Buckinghamshire, UK. Tel.: 0494 41181*

*Automated organic synthesis library*

SYNLIB (Beckman) offers high-speed retrieval of chemical syntheses that match the user structure of substructure. Searches can be broadened or narrowed by selecting loose or precise search boundaries based on chemical structure, reagents and reaction conditions. A simple graphical method enables rapid drawing of chemical structure diagrams using a low-cost CRT and lightpen. All structures are displayed in standard structural notation.

SYNLIB contains a master library of 12 000 reactions abstracted from scientific literature; an update service is also available. Internal proprietary libraries are easily prepared, maintained and searched. Search boundaries are lightpen selectable and enable the searching of the entire information content of each library entry: yields, steps, reagents and reaction conditions. *Details from Beckman (above).*

**Circle No. 15 on Reader Enquiry Card**

### Mettler in Britain

For many years Fisons Scientific Equipment Division and Mettler Instrumente AG have worked closely together to build and consolidate the laboratory balance market in the UK. Now Mettler are coming to Britain to share the growth in the industrial sector.

From 1 January 1985 a new British Company, Mettler Instruments Ltd, will import and supply Mettler and Sauter precision balances and scales to the industrial market: *Mettler Instruments Ltd, Kingsmead House, Abbey Barn Road, High Wycombe, Buckinghamshire HP11 1QW.*

Gallenkamp, MSE and the Divisional Service Organisation, the Fisons companies most closely associated with Mettler, will continue to work with Mettler and their new UK company. Gallenkamp will carry on serving the laboratory and scientific market as Mettler's principal UK distributor. MSE Scientific Instruments, who design and manufacture an extensive range of laboratory centrifuges as well as marketing other imported laboratory products, will continue to be the sole UK distributor for Mettler titration and thermal analysis equipment. And Divisional Service Organisation, the largest specialist service organization in Europe, will still provide laboratory equipment engineering support.

**Circle No. 16 on Reader Enquiry Card**

### Asbestos detection

Harnessing the power of infra-red diffuse reflectance has proved a useful feature of Pye Unicam's PU 9510/PU 9520 series of ratio recording infra-red spectrophotometers. Their low-transmission performance enables them to yield diffuse reflectance spectra from very weak signals.

The study of surfaces by diffuse reflectance has been carried out very successfully for a number of years using UV/visible spectroscopy, but only recently have accessories been available which extend the technique to the infra-red region. As the amount of radiation diffusely reflected is invariably low – around 10% or less – a ratio recording spectrophotometer must be employed. If the IR diffuse reflectance accessory is used in conjunction with any Pye Unicam IR spectrophotometer, all of which utilize the ratio recording principle, it has been found that the technique will succeed with very demanding samples without requiring much, if any, sample handling.

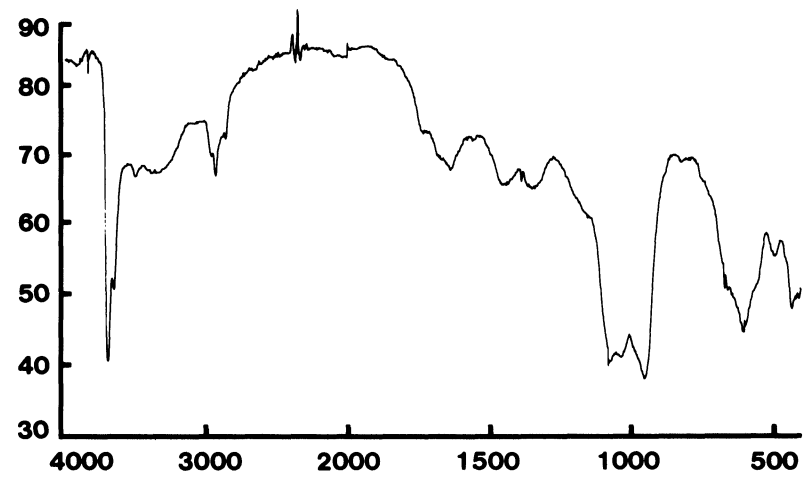

*Figure A – Sample spectrum with background correction.*

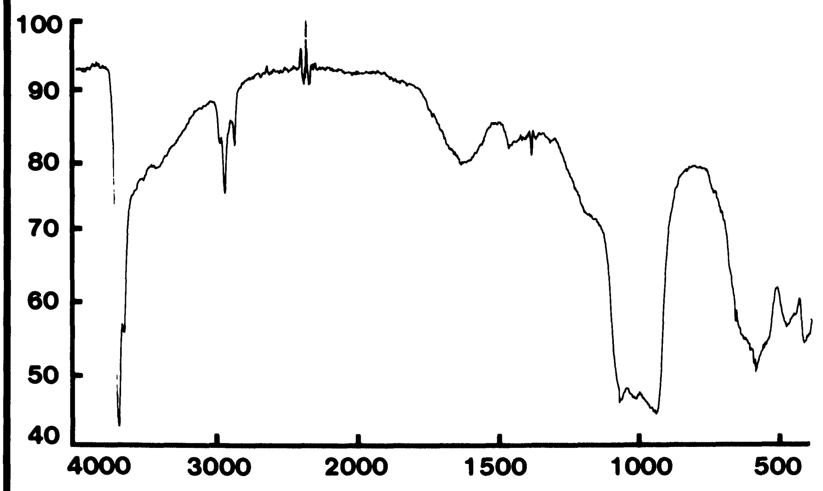

*Figure B – Spectrum of known chrysotile sample.*

*Example of asbestos detection by infra-red diffuse-reflectance spectroscopy. The low-transmission performance of Pye Unicam's PU 9510/9520 series of ratio recording IR spectrophotometers enables them to yield good diffuse-reflectance spectra from very weak signals.*

A recent example of this involved a few tiny fibres of fire-resistant material which needed checking for the presence of asbestos. The conventional approach is to grind the sample with an alkali halide and prepare microdisks – a technique demanding considerable expertise. Using diffuse reflectance, the fibres were simply placed on a bed of ground potassium bromide in the accessory sample cup and the spectrum scanned. Shown here (A) is a sample of the results, with the accessory spectrum removed by background correction. The presence of asbestos was confirmed by scanning a known sample of chrysotile by diffuse reflectance (see B) which gave a spectrum almost identical with that of the sample.

*More information from Pye Unicam Ltd, York Street, Cambridge CB1 2PX, UK. Tel.: 0223 358866.*

**Circle No. 17 on Reader Enquiry Card**

### Immunological test kits

Test kits for detection of Bordetella Pertussis lgG, lgA and lgM antibodies, baaed on solid phase enzyme immunoassay, automatic phorometric reading and relatively simple lab. work, are available from Lab-

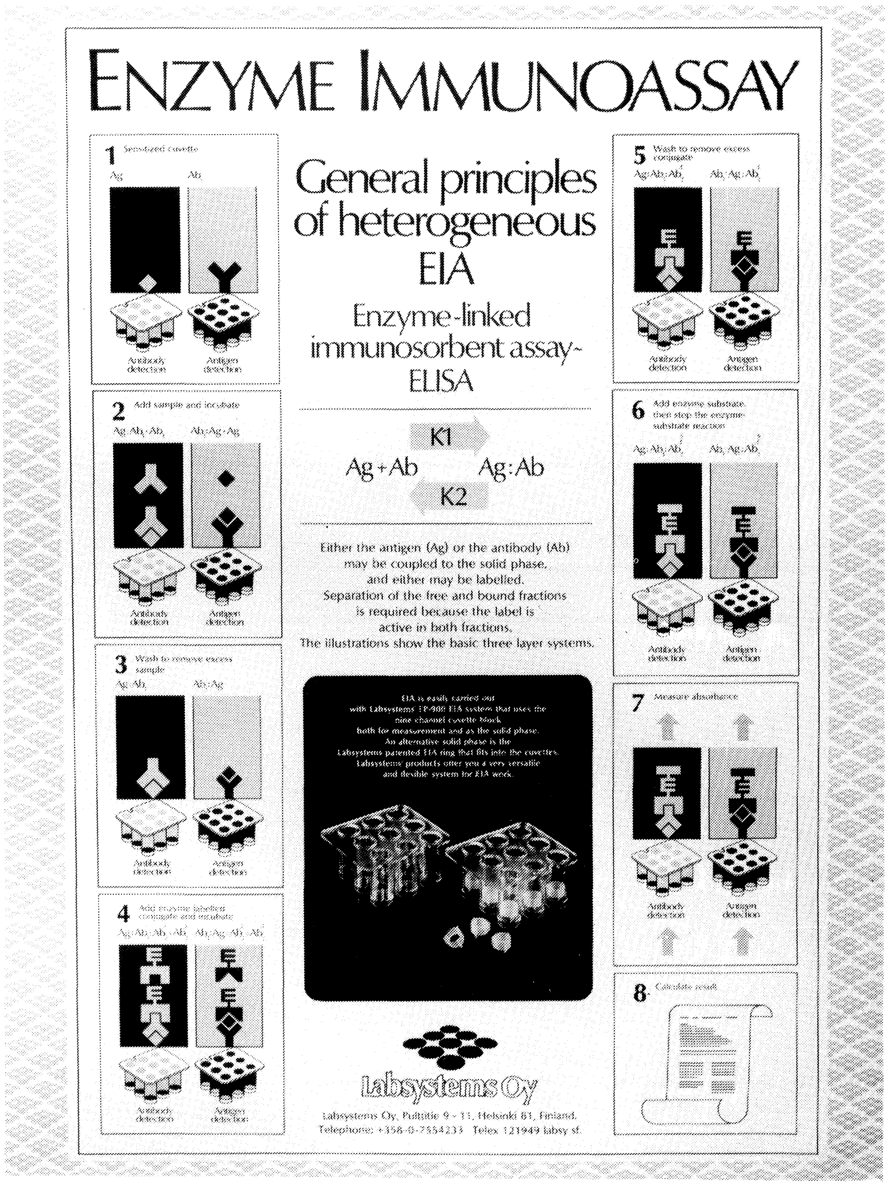

ELISA WALL CHART FROM LABSYSTEMS

*A two-colour enzyme immunoassay wall-chart, which illustrates the general principles of heterogeneous enzyme-linked immunosorbent assay (ELISA), is offered free by Labsystems. The chart (65 cm × 95 cm) describes the step-by-step procedure of both Ag and Ab detection by the ELISA technique. Copies from Labsystems (address above).*

systems. Bordetella Pertussis may not be suspected in vaccinated children and adults until they have transmitted the disease to a young infant who develops a classic and often serious illness. Precise diagnosis of Pertussis is important for effective treatment of patients, for isolation of unvaccinated infants at risk, and for differentiation of Pertussis from atypic respiratory diseases and chronic infection.

Conventional diagnostic methods are difficult to perform, slow or unreliable. Bordetella Pertussis can be isolated from only 10%–44% of patients, most successfully from infants in the acute stage of the disease. The conventional test is less reliable in convalescing patients and isolation is particularly difficult from vaccinated subjects. On the other hand, diagnosis by conventional serology is obtained too late for successful treatment or isolation. Labsystems Immunological Test Kits provide a better method for the detection of Bordetella Pertussis IgG, IgA and IgM antibodies.

*Further information from Labsystems (UK) Ltd, 12 Redford Way, Uxbridge, Middlesex UB8 1SZ, UK. Tel.: 0895 38421.*

**Circle No. 18 on Reader Enquiry Card**

### Astra Systems enzymes

Beckman Instruments, Inc. has available a package of technical performance data on the ASTRA Systems Enzyme tests. The Enzyme System includes the six most requested clinically significant enzyme tests – AST, ALT, CK, AP, LD and GGT. A series of bulletins provides data on correlation analyses with other methods, reproducibility, linearity and an assessment of the Out of Range Detection and Correction (ORDAC) feature unique to ASTRA.

*The bulletin (6115A) is available free of charge from Beckman Instruments, Inc., 2500 Harbor Blvd, Box 3100, Fullerton, California 92634, USA. Tel.: 714 871 4848.*

**Circle No. 19 on Reader Enquiry Card**

### Haematology analysers

Coulter Counter Models M2 and M4, launched towards the end of 1984, give results in as little as 12s. The results from these new instruments are provided by the Coulter Principle, already proven world-wide in thousands of applications. Both models have been designed to accommodate the total needs of the smaller laboratory, or to act as back-up and on-call systems for larger laboratories.

In terms of performance, the Coulter Counter Model M2 offers the two most widely requested haematology parameters: White Blood Cell Count (WBC) and Haemoglobin (Hb). The Model M4 performs a four-parameter profile consisting of WBC, Hb, Red Blood Cell Count (RBC) and Haematocrit (Hct).

*For further information contact Coulter Electronics Ltd, Northwell Drive, Luton, Bedfordshire LU3 3RH, UK. Tel.: 0582 582442.*

**Circle No. 20 on Reader Enquiry Card**

